# Supplementary material for: Do We Need Public Green Spaces Accessibility Standards for the Sustainable Development of Urban Settlements? The Evidence from Wrocław, Poland
Source: Int J Environ Res Public Health. 2023 Feb 9;20(4):3067. doi: 10.3390/ijerph20043067 (PMC9960964; doi:10.3390/ijerph20043067)
Supplement: Supplementary file 1 [file ijerph-20-03067-s001.zip › ijerph-2174229-supplementary.pdf]

Table S1. Characteristics of public green spaces (PGS) localised in the study area and the buffer zones.

Type of PGS	No	Surface [ha]	Name	Orthophotomap with PGS localisation	Short characteristic	Facilities							
						Paths	Seats: benches or other elements	Play-grounds	Outdoor gyms, fitness equip-ment	Sport fields	Multifun-ctional areas for picnics or/and cultural and sport activities	Sculptu-res and elements of park architectu-re: pergolas, gazebos, bridges, fountains	Food outlets: restaurants, cafés, bars, kiosks
1	2	3	4	5	6	7	8	9	10	11	12	13	14
**PGS I**													
PGS I	1	0.07	Square at Pawia street	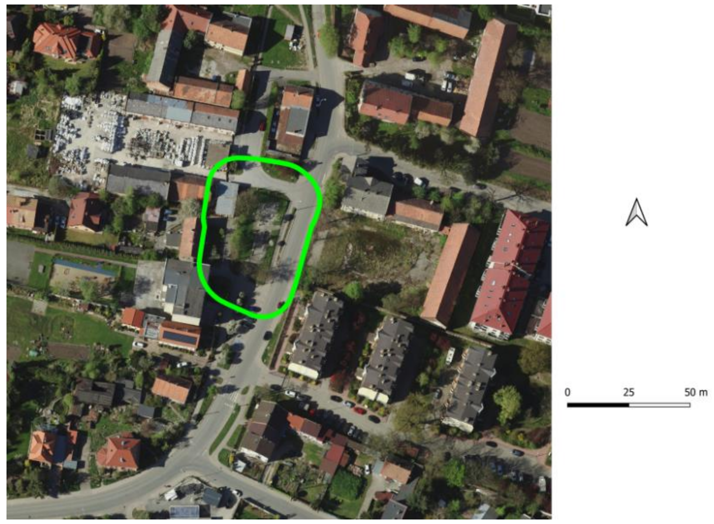	Located in the Wojszyce housing estate in the place of the historical centre of the old village, transformed into a public green space with an outdoor gym in 2017 (initiative of the residents of the estate).	+	+	–	+	–	–	–	–
PGS I	2	0.12	Square at Kosmiczna street	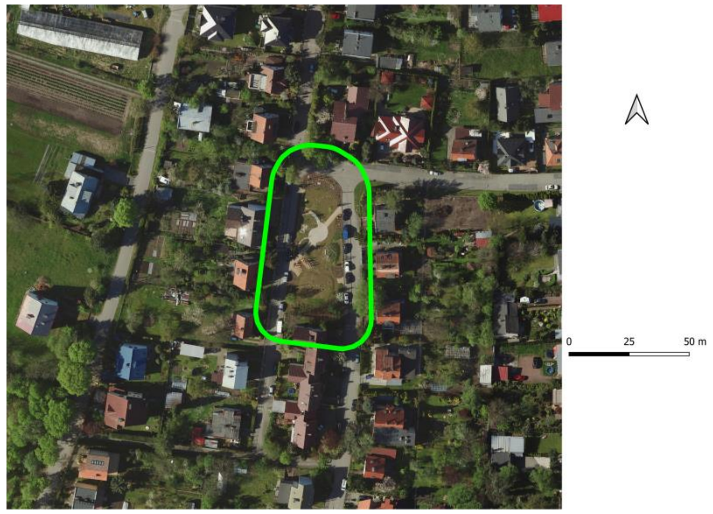	Located in the Wojszyce housing estate, established together with single-family housing from the turn of the 1970s and 1980s, upgraded in 2021 (initiative of the residents of the estate).	+	+	+	–	–	–	+	–

1	2	3	4	5	6	7	8	9	10	11	12	13	14
**PGS I**	**3**	**0.19**	Square at Zawiszy Czarnego and Kwitnąca streets	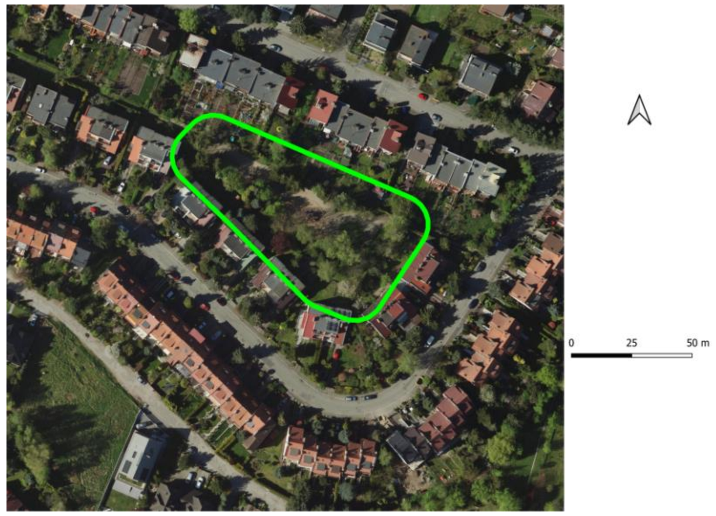	Located in the Wojszyce housing estate, established together with single-family housing development (terraced houses) from the turn of the 1970s and 1980s, upgraded in 2020 (initiative of the residents of the estate).	+	+	+	—	—	—	—	—
**PGS I**	**4**	**0.22**	Square at Biwakowa street	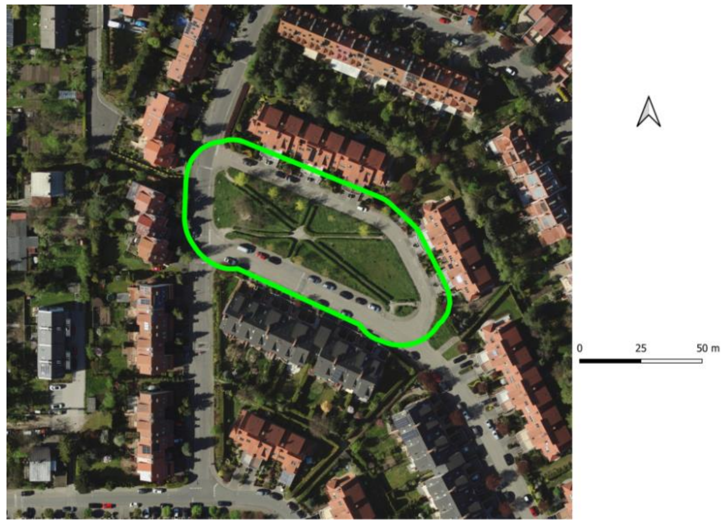	Located in the Wojszyce estate, established together with single-family housing development (terraced houses) in the first decade of the 21st century.	+	—	—	—	—	—	—	—
**PGS I**	**5**	**0.30**	Square at Skibowa Street	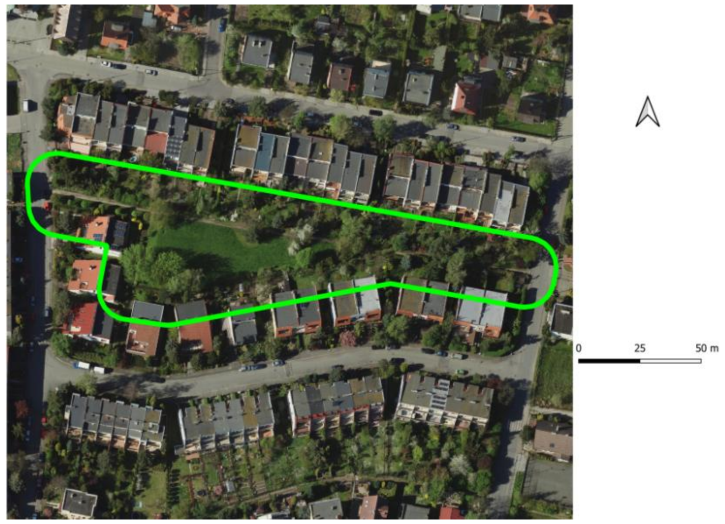	Located in the Wojszyce estate, established together with the single-family housing development (terraced and semi-detached houses) from the turn of the 1970s and 1980s.	+	+	—	—	—	+	—	—

1	2	3	4	5	6	7	8	9	10	11	12	13	14
**PGS I**	**6**	**0.32**	Square at Parafialna street	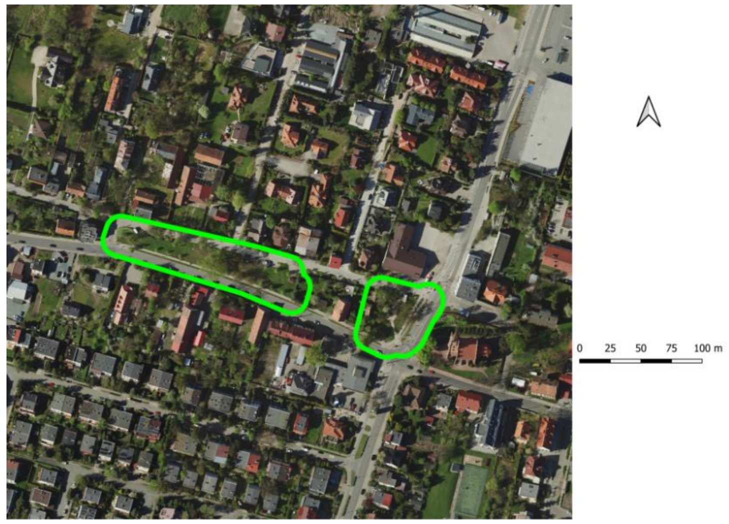	Located in the Ołtaszyn housing estate, established in the old centre of the historic village, near the church, renovated in 2020 (initiative of the residents of the estate).	+	+	—	—	—	—	—	—
**PGS I**	**7**	**0.51**	Square at Nefrytowa street	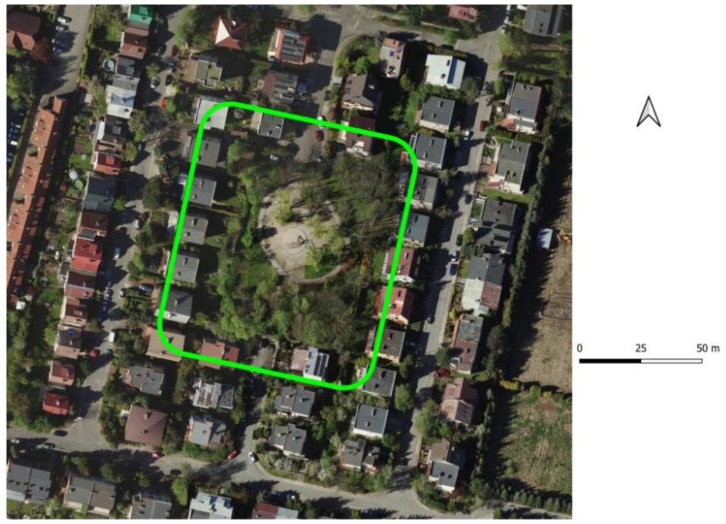	Located in the Ołtaszyn housing estate, established together with single-family housing development from the turn of the 1970s and 1980s, partly renovation in 2014 in 2022 (initiative of the residents of the estate).	+	+	+	—	—	—	—	—
**PGS I**	**8**	**1.09**	Wojszycki mini park	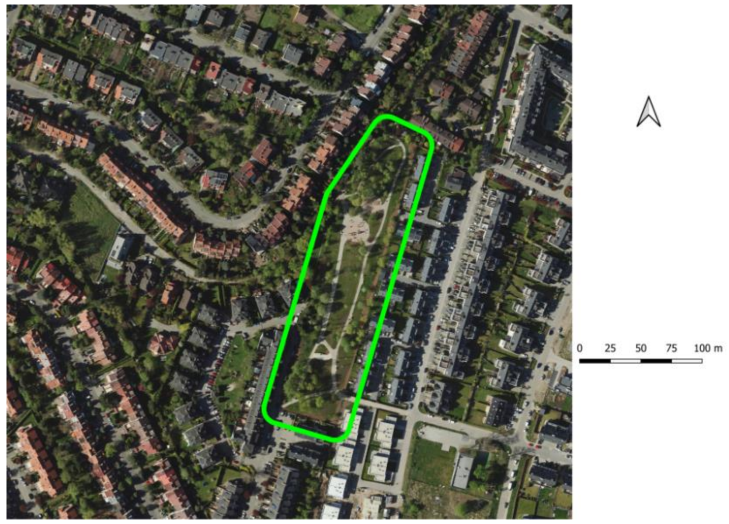	Located in the Wojszyce housing estate, established as a new public green spaces in 2018 (stage I), 2019 (stage II). (initiative of the residents of the estate)	+	+	+	—	—	+	—	—

1	2	3	4	5	6	7	8	9	10	11	12	13	14
**PGS I**	**9**	**1.17**	Gen. T. Rozwado-wski mini park	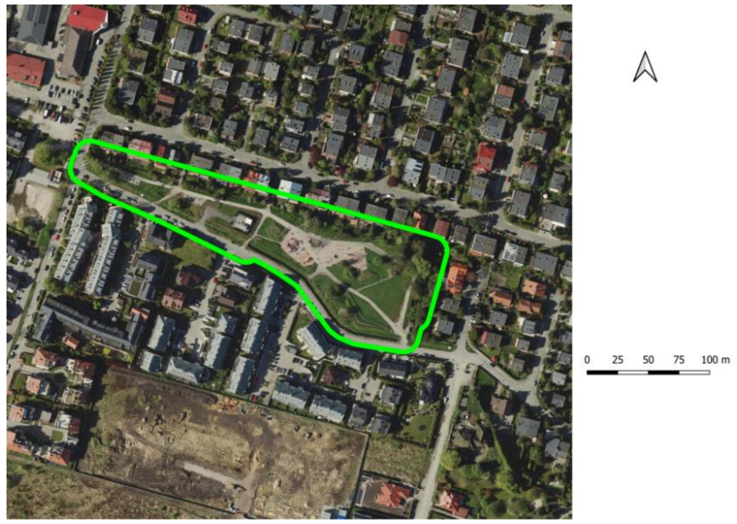	Located in the Ołtaszyn housing estate, established as a new public green spaces in 2016-2018 (initiative of the residents of the estate).	**+**	**+**	**+**	**+**	**—**	**+**	**—**	**—**
**PGS II**													
**PGS II**	**10**	**2.4**	Neighbor-hood park „Zielony Gaj”	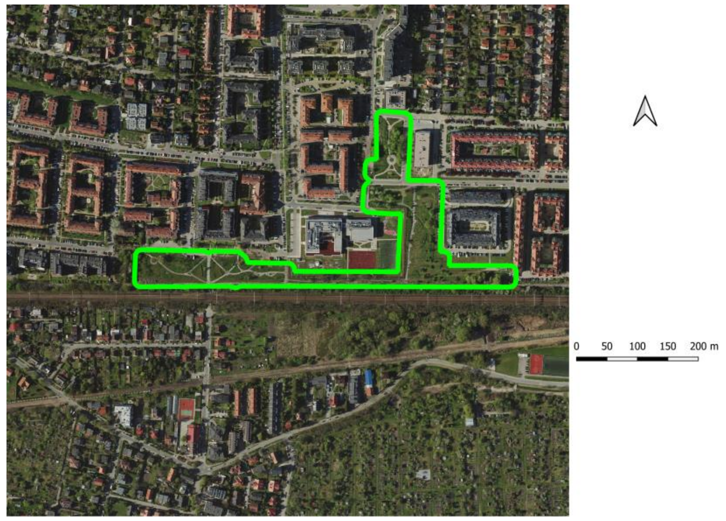	Located in the buffer of the examined housing estates, established as a park in 2018 on the place of informal green space (initiative of the residents of the estate).	**+**	**+**	**+**	**+**	**—**	**+**	**—**	**—**
**PGS II**	**11**	**2.25**	Neighbor-hood park at Bardzka street	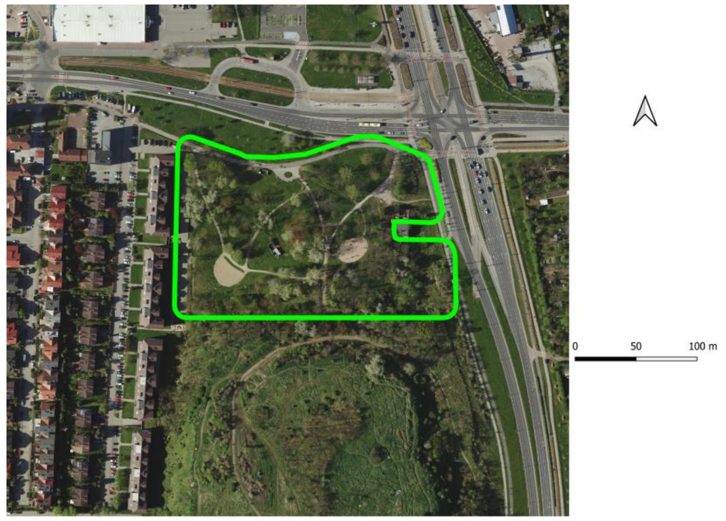	Located in the buffer of the examined housing estates, established in 2016-2017 on the place of informal green space (initiative of the residents of the estate).	**+**	**+**	**+**	**—**	**—**	**+**	**—**	**—**

1	2	3	4	5	6	7	8	9	10	11	12	13	14
**PGS II**	**12**	**3.68**	Promenade at Koszycka street	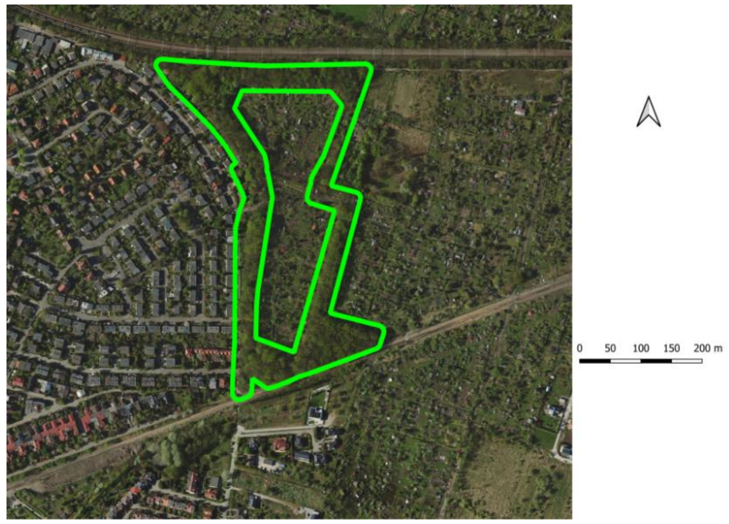	Located in the Ołtaszyn housing estate, a fragment of the historic promenade from the beginning of the 20th century, only part open to the public, requires restoration and revalorisation work	+	—	—	+	—	—	—	—
**PGS III**													
**PGS III**	**13**	**5.06**	„Solar Park"	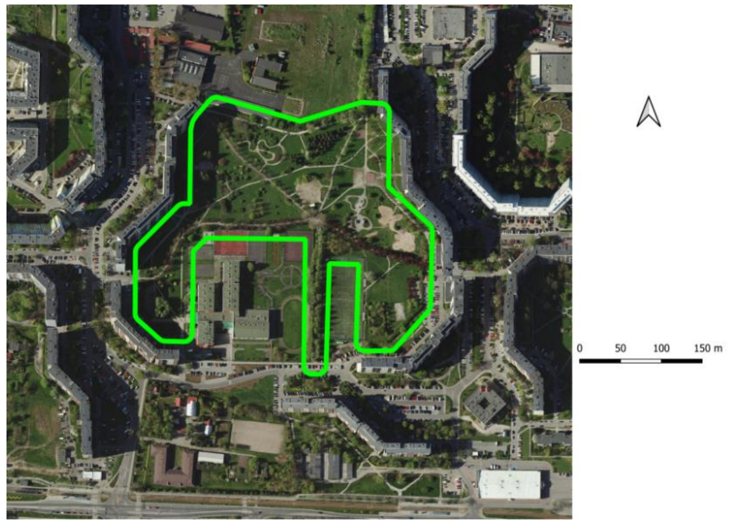	Located in the buffer of the examined estates, surrounded by multi-family buildings from the 1970s and 1980s., partially renovated in the second decade of the 21st century.	+	+	+	—	—	+	—	—
**PGS III**	**14**	**5.62**	„Tarnogajski Park"	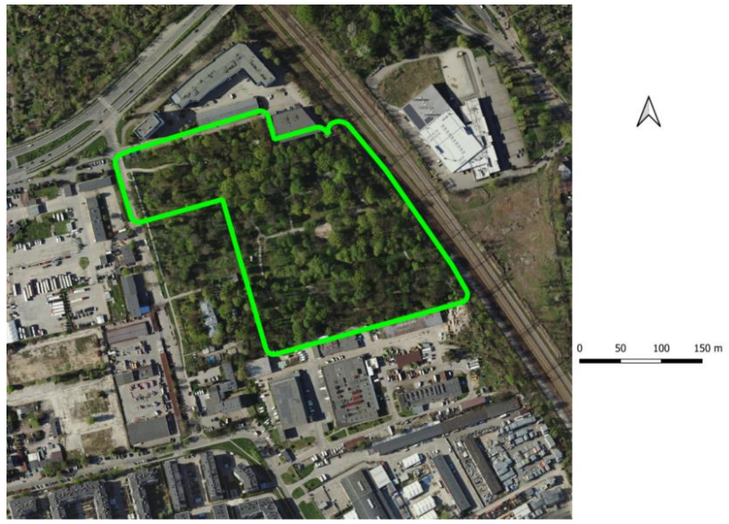	Located in the buffer of the examined housing estates, established on the place of historical cemetery, open in 2018. (initiative of the residents of the estate).	+	+	+	+	—	+	—	—

1	2	3	4	5	6	7	8	9	10	11	12	13	14
**PGS III**	**15**	**12.5**	„Brochowski Park"	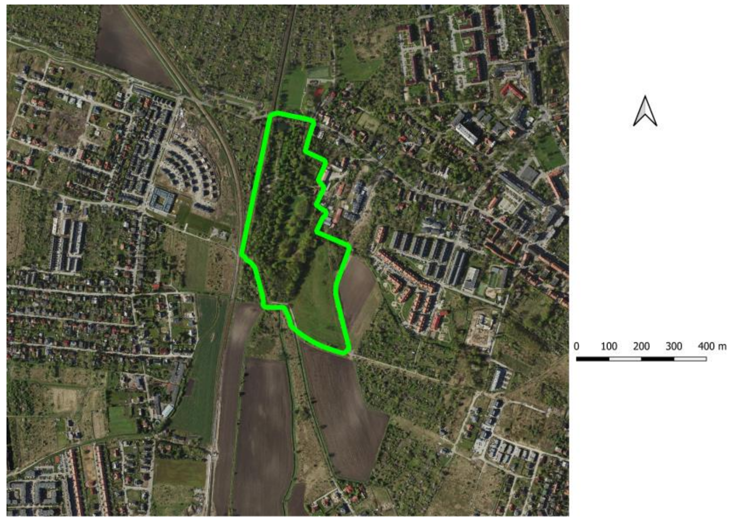	Located in the buffer of the examined housing estates, historic old park, renovated and enlarged in the second decade of the 21st century.	+	+	+	+	+	+	+	+
**PGS III**	**16**	**23.28**	„South Park"	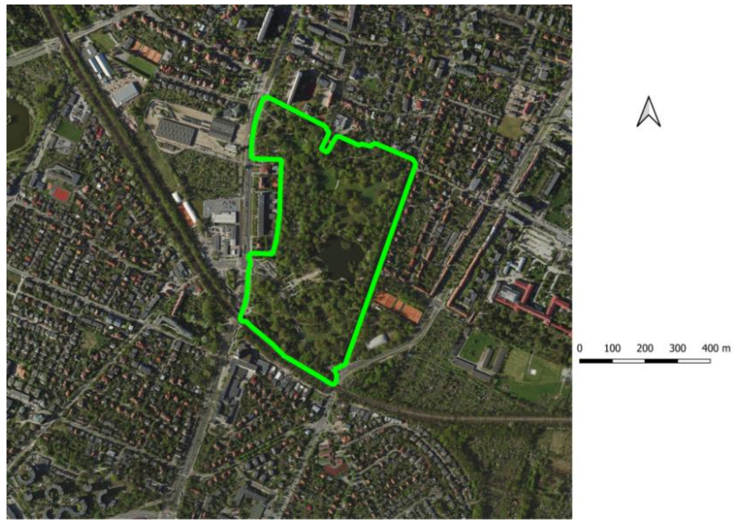	Located in the buffer of the examined housing estates, historic park created in the second half of the 19th century.	+	+	+	+	+	+	+	+
**PGS III**	**17**	**24.71**	„Skowroni Park"	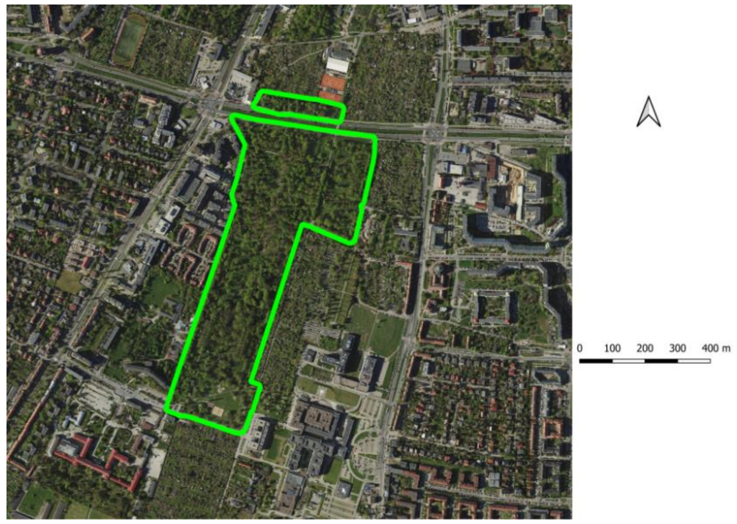	Located in the buffer of the examined housing estates, established in the place of a historical cemetery. Open in 1967.	+	+	+	+	+	+	—	—
